# A Single-Center, Blinded, Placebo-Controlled Study Evaluating Cosmetic Efficacy and Safety of a Novel Topical GLPSGLT in Glucagon-Like Peptide-1 Analog-Treated Patients

**DOI:** 10.1093/asjof/ojaf030

**Published:** 2025-04-25

**Authors:** Alec Semersky, Sydney Pryor, Caitlin Barwood-Parent, Mike Lee, Karen Copeland, Julius Few

## Abstract

Glucagon-like peptide-1 receptor agonists (GLP-1 RAs) and Sodium-glucose cotransporter 2 (SGLT-2) inhibitors are widely used for Type 2 diabetes and weight management but may adversely affect skin quality, resulting in dermal thinning, decreased elasticity, and accelerated aging—a phenomenon referred to as “GLP-1 Face.” A novel topical serum, GLPSGLT (Aforé LLC), was developed to counteract these effects using a proprietary blend of a bioavailable retinoic acid derivative, peptides, and botanical agents to support keratinocyte function and dermal repair. The authors of the study aim to evaluate the safety and efficacy of GLPSGLT serum in improving facial skin quality in patients undergoing GLP-1 RA or SGLT-2 therapy. In this split-face, double-blind pilot study, 7 female patients (median age 55) on stable GLP-1 RA/SGLT-2 therapy applied GLPSGLT serum to one side of the face and placebo to the other, twice daily for 6 weeks. Assessments at baseline, Day 21, and Day 42 included the Global Ranking Scale (GRS), standardized photography reviewed by a blinded physician, and a 26-item patient-satisfaction questionnaire. Treated sides showed statistically significant improvements across all 13 GRS domains vs placebo (*P* < .0001), particularly in hydration, surface roughness, pigmentation, vasculature, visible pores, and static wrinkles. The blinded reviewer correctly identified the treated side in all cases. Patients reported greater satisfaction with skin texture, firmness, radiance, and hydration. No adverse events were reported. GLPSGLT serum significantly improved skin quality in GLP-1 RA/SGLT-2-treated patients and was well-tolerated, warranting further investigation in larger, histologically assessed cohorts.

**Level of Evidence: 4 (Therapeutic):**

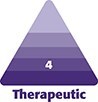

Glucagon-like peptide-1 receptor agonists (GLP-1 RAs) are a popular class of drugs that are FDA approved to treat Type 2 diabetes as well as obesity and cardiovascular disease prevention.^1^ Because more GLP-1 RAs come to market with decreased prices, simpler use, and at-home availability through telehealth providers, the percentage of GLP-1 RA–treated patients in aesthetic plastic surgical settings is likely to increase. Although the safety profile of GLP-1 RAs is favorable, the drugs do have side effects recognized in the medical literature, including gastrointestinal distress, pancreatic concerns, cardiovascular effects, and injection-site reactions.^2^ Some investigators are beginning to look at the effects of GLP-1 RA on adipose and skeletal muscle, whereas we theorize a major source of pathology for what we refer to as “GLP Face” and “GLP Skin” is the result of dermal impairment.^3^

Based on anecdotal findings in facelift surgery, we hypothesize that there is a direct link between GLP-1 treatment and dermal/epidermal impairment. In vitro and in vivo studies have demonstrated GLP-1 RAs suppressing keratinocyte proliferation, suppressing cytokine production, reducing skin structural integrity and barrier function, and reducing epidermal thickness.^4-6^

Glucocorticoids and systemic steroids serve as an effective treatment for hyperproliferative skin conditions, such as hypertrophic scarring, by helping to suppress inflammation and reduce keratosis.^7^ By suppressing keratinocyte proliferation and migration, these treatments can effectively manage certain inflammatory skin conditions that cause excess keratosis, like psoriasis.^8^ Glucocorticoid administration for variable term is shown to inhibit keratinocyte and fibroblast activity at the cellular and cytokine level.^9,10^ These changes have led to increased skin susceptibility to ultraviolet damage, decreased elasticity, decreased barrier function, increased transepidermal water loss, and increased fragility.^11,12^

Use of GLP-1 RAs may result in similar skin changes to those observed in patients on systemic steroids and glucocorticoids, because of similar antiproliferative effects on keratinocytes, macrophages, and fibroblasts. Just as glucocorticoids and systemic steroids reduce keratinocyte proliferation, so too do GLP-1 RAs.^6,9^ Keratinocytes have been found to exhibit GLP-1 receptors, and their proliferation is modulated by GLP-1 RAs—liraglutide specifically.^6^ In studies examining liraglutide's effects on keratinocytes, it was found both in vitro and in vivo that liraglutide suppressed keratinocyte proliferation.^5,6^ Similar to glucocorticoids, liraglutide has been proposed as a potential treatment for psoriasis, given its antiproliferative effects.^4^ Taken together with the observed accelerated aging, and textural differences observed during facelift surgery on GLP-1 RA–treated patients, it is hypothesized that long-term use of GLP-1 RAs may lead to skin atrophy through a similar mechanism to glucocorticoids and systemic steroids. In the burn literature, it is known that patients with large surface area burns on systemic steroid therapy have taken large doses of systemic Vitamin A, which partially reverses steroid-induced transforming growth factor beta 1 and insulin-like growth factor 1 suppression, thus protecting the dermis and epidermis from classic steroid changes.^13^

To our knowledge, our theory represents the first time an analogy has been made between glucocorticosteroid effects on the skin and GLP-1 RA/Sodium-glucose cotransporter 2 (SGLT-2) effects on the skin, thus identifying an important consequence of GLP-1 RA/SGLT-2 treatment on the skin and a potential method to combat it.

In the current prospective study, a novel topical GLPSGLT l serum (Afore LLC, Chicago, IL) was tested for safety and efficacy on facial skin in a cohort of GLP-1 RA–treated patients. The formulation was designed to provide a bioavailable local form of retinoic acid to the dermis, theoretically helping to partially reverse cellular and cytokine disruption.^14^ This study represents the first placebo-controlled study to evaluate a topical serum used to enhance the elasticity, barrier function, and textural changes seen in the GLP-1 RA patient.

## METHODS

### Study Design

This single-center, split-face, placebo-controlled, blinded pilot study assessed the clinical effects of a novel topical serum (GLPSGLT topical serum) when applied twice daily to facial skin. The GLPSGLT serum contains a proprietary blend of water, retinoic acid, peptides, sodium hyaluronate, and botanically derived ingredients. The placebo-controlled topical in this study was designed to look, feel, and smell identical to the GLPSGLT serum, and its ingredients were as follows: water, aloe vera gel, sodium hyaluronate, phenoxyethanol, and potassium sorbate. This study was approved by the Advarra IRB before initiation.

### Statistical Methods

All data analysis was performed using JMP Pro 18 (JMP Statistical Discovery LLC, Cary, NC). Matched pair tests were used to test for treatment effects. Unadjusted *P*-values for both parametric (*t* test) and nonparametric (signed-rank) assessments are provided.

A total of 7 patients were enrolled; demographics are shown in Table 1. Patients were selected who met inclusion criteria based on skin quality related to GLP-1 analog therapy, SGLT analog therapy, and aging. The inclusion criteria were healthy females or males of ages between 18 and 75 years receiving GLP-1 analog or SGLT-2 therapy for a minimum of 30 days before the start of the study. The inclusion criteria for skin quality were presenting with at least one of the following: volume loss, loss of elasticity, presence of fine lines and wrinkles, enlarged pores, photodamage, surface roughness, and uneven pigmentation. Patients who had received botulinum toxin A, filler, or energy-based treatments within 3 months of study enrollment or who had undergone rejuvenation procedures to the face or neck (eg, facelift surgery, microneedling, microdermabrasion, and chemical peels) within 30 days of the start of the study were excluded. Those who were current smokers, had a history of heavy smoking, had uncontrolled systemic inflammatory conditions, or had active dermatologic conditions in the areas to be treated were also excluded. For the duration of the study, patients did not receive any aesthetic treatments on their face or neck and limited their use of topical products to the product under study, the placebo topical, a gentle facial cleanser, a daily facial moisturizer, and routine 25% zinc-oxide-based SPF 30 sunscreen application daily as recommended by the American Academy of Dermatology. The gentle facial cleanser, daily facial moisturizer, and routine 25% zinc-oxide-based SPF 30 sunscreen were provided to patients at no cost.

**Table 1.** Patient Characteristics (*n* = 7)

Characteristic	Value
Median age, years (range)	55 (34-64)
GRS skin type, *n* (%)	
I: pale white or freckled	5 (71.4)
II: white	0 (0.0)
III: white to light brown	0 (0.0)
IV: moderate brown	1 (14.3)
V: dark brown	1 (14.3)
VI: very dark brown to black	0 (0.0)
GLP-1 or SGLT drug, *n* (%)	
Tirzepatide	3 (42.8)
Semaglutide	3 (42.8)
Dulaglutide	1 (14.3)

GLP-1, glucagon-like peptide-1; GRS, Global Ranking Scale.

In this pilot study, the patients served as their own placebo-control group using a split-face study design. Following baseline assessment, each patient was provided with 2 bottles of product in unbranded amber glass containers with pumps: 1 labeled “LEFT” by the product manufacturer and 1 labeled “RIGHT” by the product manufacturer. Patients were instructed to apply a thin layer of the serum labeled LEFT to the left side of their face following use of the provided mild facial cleanser on the entire face in both the morning and evening. After applying the topical labeled LEFT, patients were instructed to wash their hands and then apply a thin layer of topical labeled RIGHT to the right side of their face. In the mornings, patients were instructed to apply a thin layer of the daily facial moisturizer to both sides of the face, followed by a thin layer of the 25% zinc-oxide-based sunscreen, after applying both LEFT and RIGHT topicals. This regimen was continued for 42 days (6 weeks) with follow-up visits at Days 21 and 42.

Compliance was ensured by weighing the product containers at the end of the study. Patients also filled out a diary in which they recorded each application of product, as well as recorded any changes to their skin appearance and perceived side effects. Patients were not reimbursed for their time, but the cost of parking at the study facility was covered by the senior author's private practice.

### Efficacy and Safety Measures

#### Primary Endpoint

**Figure 1.**
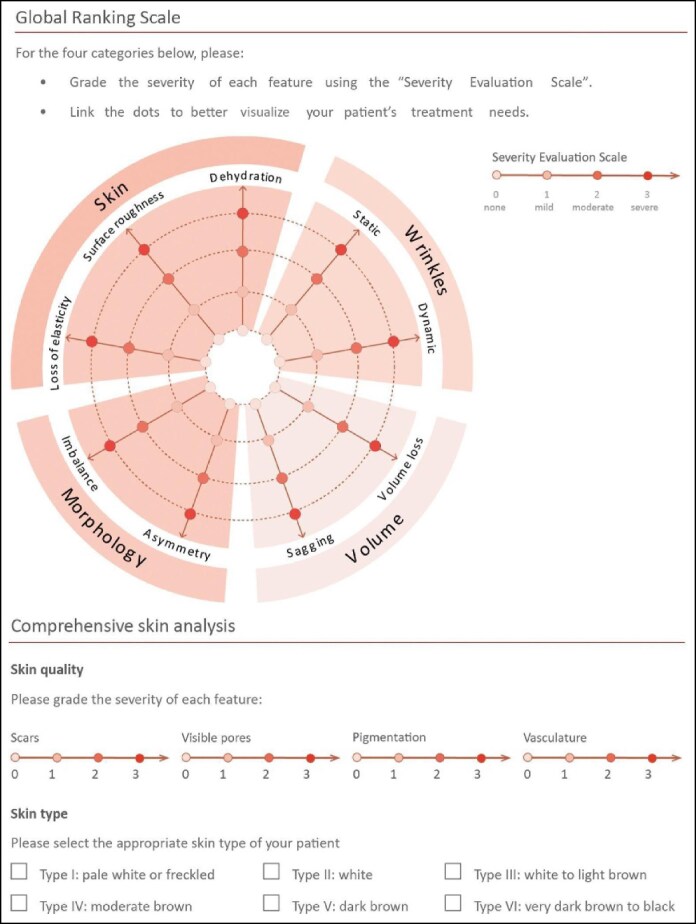
Global Ranking Scale and comprehensive skin analysis. This figure originally appeared within Jain et al^15^ published by Wiley under a license type CC BY-NC license agreement, which permits reproduction of the image with proper attribution to the original work.

The evaluators, clinical staff, and patients were blinded to placebo vs active study product. An off-site clinical coordinator, not involved in the assessment, was the control monitor. At baseline and each follow-up visit, standardized photographs of the treatment areas were captured. Patients were assessed live (co-assessment included both the patient and the treating clinician) using the Global Ranking Scale (GRS; Figure 1) with comprehensive skin analysis.^15^ The primary endpoint of this pilot study was the difference in the GRS between sides of the face compared with baseline. The GRS assessments were completed for the entire face on Day 1 (baseline). In addition, the GRS was conducted separately for each side of the face on Days 21 and 42. Both patients and the investigator were blinded to the treatment vs placebo sides of the face. For each of the 13 domains included in the GRS (loss of elasticity, surface roughness, dehydration, static wrinkles, dynamic wrinkles, volume loss, sagging, asymmetry, imbalance, scar presence, visible pores, pigmentation, and vasculature) grading ranges from 0 to 3 (0, none; 1, mild; 2, moderate; and 3, severe). The patient and investigator were blinded to baseline scores and any scores from previous follow-ups at each evaluation. The protocol was based on our validated protocol from a previous published study in the *Aesthetic Surgery Journal*.^16^

#### Independent Photographic Review

In addition to the GRS, an independent review of standardized before and after images was carried out by a blinded board-certified plastic surgeon working in a remote clinic near the study site. The independent reviewer, who was blinded to the patient GRS scores for both the treatment and placebo sides of the face, was provided with patient images at Day 1 (baseline) and Day 42, and asked to select which side (left or right) showed significant improvement between Days 1 and 42, or select “no difference” if no qualitative difference was observed. The evaluator was asked to only review and select a side for domains that could be assessed in a remote setting: static wrinkles, scars, vasculature, surface roughness, and pigmentation. The remaining GRS domains were excluded: dynamic wrinkles, loss of elasticity, sagging, volume loss, asymmetry, and dehydration. The percentage of patients considered improved in each domain was then calculated.

#### Secondary Endpoint

A 26-question patient-satisfaction questionnaire was completed independently by patients, at home, before their office visits on Days 21 and 42. The questionnaires included questions addressing functional outcomes and satisfaction, as well as several questions that overlap with GRS skin quality domains (Appendix).

#### Safety

Safety was monitored through a virtual visit on Day 3 and at each follow-up visit (Days 21 and 42) through assessment of the facial skin by the investigator and by review of patient diaries. This study was approved by an IRB (Advarra IRB, Columbia, MD) and adhered to the standards set forth in the World Medical Association's Declaration of Helsinki. Written consent was provided, by which the patients agreed to the use and analysis of their data and publication of their photographs.

## RESULTS

The study took place from January to March of 2025, and enrolled 7 patients (all female) with a median age of 55 years (range, 34-64 years; Table 1). Participants were using various GLP-1 RA medications, including 3 patients (42.8%) using tirzepatide (Zepbound; Eli Lilly and Company, Indianapolis, IN), Mounjaro (Eli Lilly and Company), 3 patients (42.8%) using semaglutide (Ozempic; Novo Nordisk, Copenhagen, Denmark), Wegovy (Novo Nordisk), and 1 patient (14.3%) using dulaglutide (Trulicity; Eli Lilly and Company). Patients were fully compliant with the completion of all GRS evaluations and patient questionnaires. Three different Fitzpatrick skin types were included in this study (Table 1). All patients maintained a stable weight within 5 pounds.

To assess the GRS, the difference from baseline was calculated for each patient at each time point for each side (ΔBL right and ΔBL left). Matched pairs were used to test whether the difference between sides was equal to zero. In all cases, the difference between the treated and untreated sides was greater than zero, indicating that the GRS scores were better for the treated side. These differences were statistically significant with both a *t* test and a signed-rank test (Table 2). A post hoc correction using a simple Bonferroni correction was considered. Using the adjusted *P*-value of 0.05/13 = 0.0038 as a threshold for significance, we still conclude that the GRS scores are better for the treated side when compared with the untreated side.

**Table 2:** Mean Differences and *P*-Values for Tests that L − R = 0 for Each Feature

	ΔBL left − ΔBL right	*t* test	Signed-rank
Category label	Mean	*P*-value	*P*-value
Morphology asymmetry	−1.5	<.0001	.0002
Morphology imbalance	−1.2	<.0001	.0002
Skin quality pigmentation	−1.4	<.0001	.0001
Skin quality scars	−0.9	<.0001	.0005
Skin quality vasculature	−1.6	<.0001	.0002
Skin quality visible pores	−1.4	<.0001	.0001
Skin dehydration	−1.6	<.0001	.0001
Skin loss of elasticity	−1.3	<.0001	.0001
Skin surface roughness	−1.9	<.0001	.0001
Volume sagging	−1.3	<.0001	.0002
Volume loss	1.2	<.0001	.0002
Wrinkles: dynamic wrinkles	−1.1	<.0001	.0002
Wrinkles: static wrinkles	−1.3	<.0001	.0001

**Figure 2.**
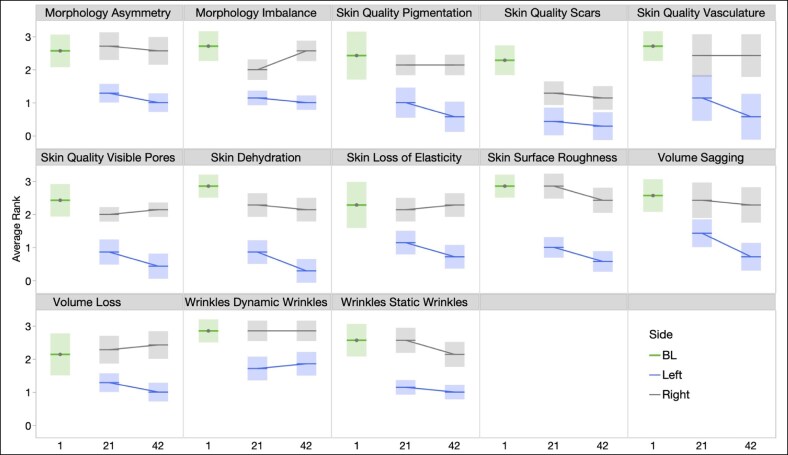
Mean Global Ranking Scale scores at baseline, Day 21, and Day 42 for left and right sides of the face.

Mean GRS scores with standard errors are shown in Figure 2 for baseline and each follow-up visit for both the treatment side and placebo side. Mean baseline scores were reflective of mild-to-moderate severity, with the most severe average scores observed for loss of elasticity, surface roughness, vasculature, dehydration, visible pores, and imbalance, as well as pigmentation. For each domain, the improvement on the treated (left) side was apparent by Day 21, and additional improvement was observed at Week 6 (Day 42). The nontreated sides (right) tended to remain unchanged from baseline.

Although an improvement was observed for each GRS domain, including those for which one would not expect a topical treatment to have an effect (ie, volume loss), assessment of the degree of improvement in each domain reveals that the greatest changes (from Day 1 to Day 42) were present in those domains consistent with topical treatments (Figure 2). Domains in which a >1-point improvement was observed include dehydration, visible pores, static wrinkles, surface roughness, pigmentation, and vasculature.

The blinded reviewer correctly identified the treatment side in 100% of patients across all domains evaluated (surface roughness, static wrinkles, vasculature, visible pore size, pigmentation, and scars).

**Figure 3.**
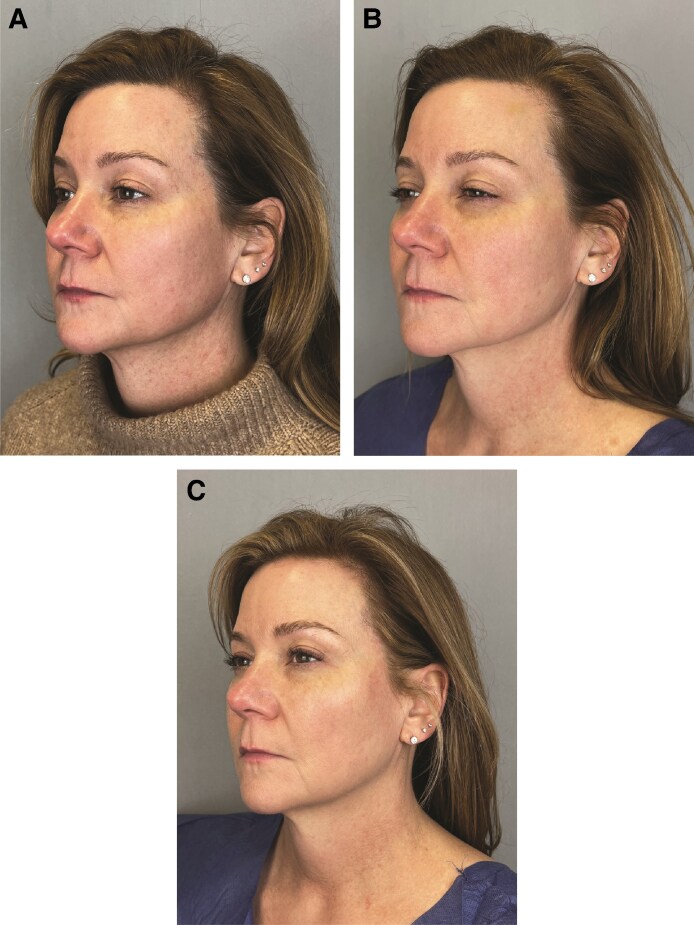
A 54-year-old female with a present history of glucagon-like peptide-1 therapy for 6 months and stable weight demonstrating the treatment left side of the face (A) at baseline, (B) at 21 days, and (C) at 42 days posttopical application of GLPSGLT serum twice daily. Changes (Δ) to Global Ranking Scale scores between baseline and Day 42 for patient were as follows: surface roughness: −2, static wrinkles: −2, visible pores: −3, pigmentation: −2, and vasculature: −2. These improvements were rated as statistically significant compared with placebo control by patient, principle investigator, and independent—blinded to treatment—plastic surgeon.

**Figure 4.**
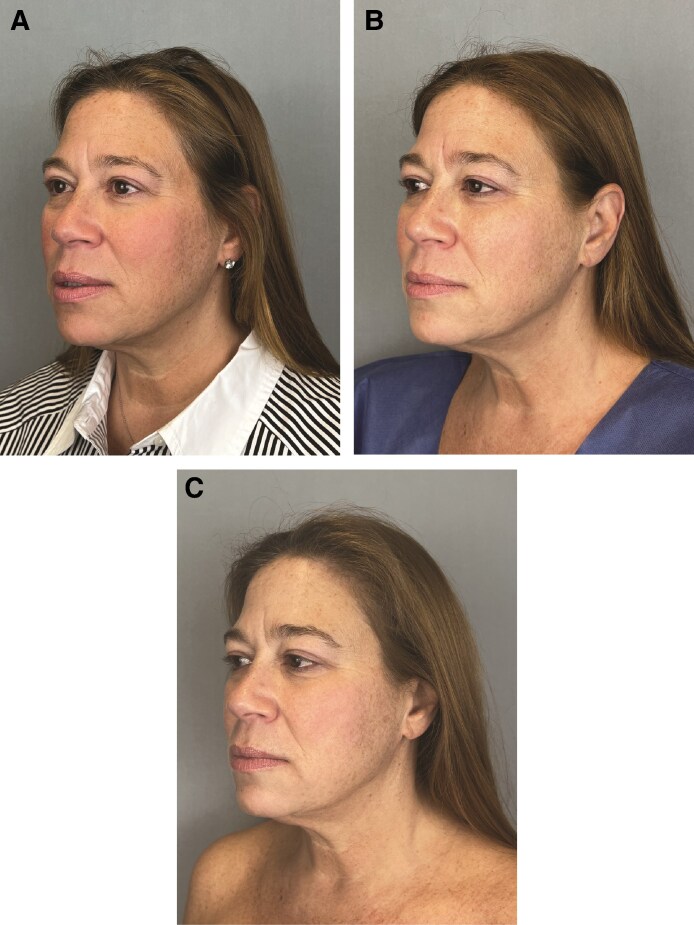
A 51-year-old female on glucagon-like peptide-1 therapy for 14 months and stable weight presenting (A) baseline for left treatment side, (B) after 21 days of twice daily application of GLPSGLT serum, and (C) after 42 days of twice daily application of GLPSGLT serum.

**Figure 5.**
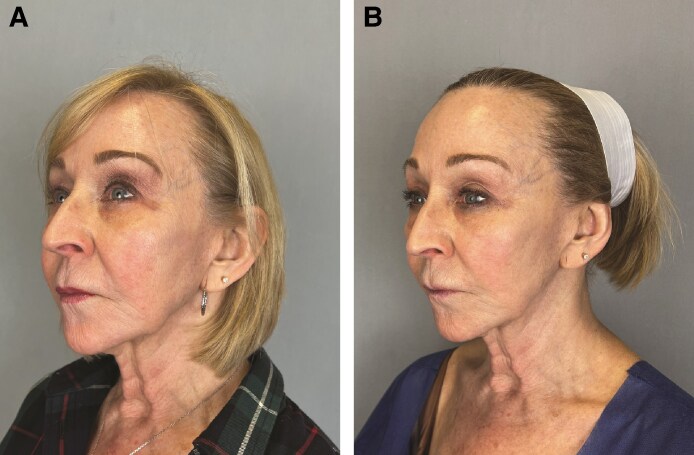
A 63-year-old female on glucagon-like peptide-1 therapy for 1 year and stable weight (A) at baseline with skin characterized by prominent fine lines and redness and (B) after 42 days of twice-daily application of GLPSGLT serum.

**Figure 6.**
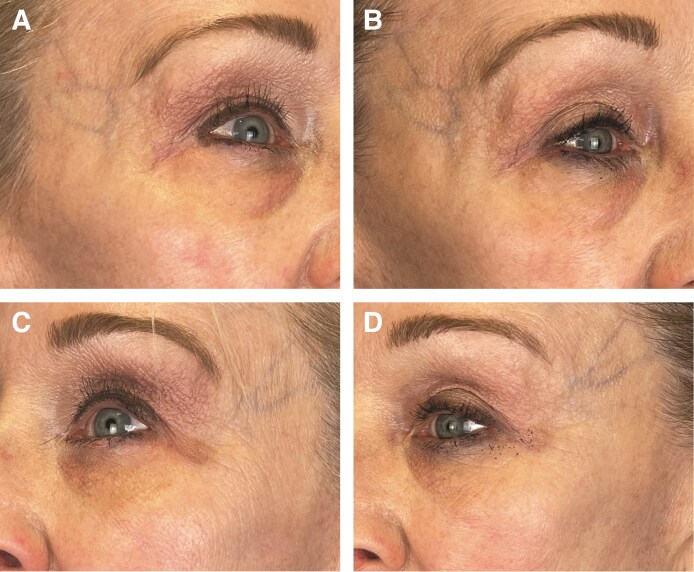
A 63-year-old female on glucagon-like peptide-1 therapy for 1 year and stable weight (A) and (C) at baseline; (B) after 6 weeks of treatment with the placebo topical; and (D) after 6 weeks of twice-daily application of GLPSGLT topical.

In Figures 3-6, patient before and after images are shown.

## DISCUSSION

The GLPSGLT topical is a unique and novel topical formulation that includes ingredients known to stimulate collagen and elastin production in the skin, increase skin cell turnover, and increase keratinocyte proliferation. To our knowledge, this represents the first study of its kind to evaluate such a combination of ingredients for both cosmetic enhancement and the reversal of drug-induced skin changes related to GLP-1 RA medications, specifically tested on GLP-1 RA–treated patients. This study provides evidence, in a small number of patients, that this combination may be effective for improving skin elasticity, surface roughness, skin texture and smoothness, dehydration, appearance of pores, fine lines and wrinkles, and pigmentation. These improvements are consistently captured across 3 different measures: the GRS scale, patient-satisfaction surveys, and independent physician review. Increased skin elasticity and firmness at Day 42 on the treated side—observed in all of the patients—presents an opportunity for future, more in-depth investigation related to histopathological changes to the skin. It is theorized that the GLPSGLT topical increased epidermal thickness, in direct contrast to observed epidermal thinning in GLP-1 RA–treated patients; however, skin biopsies and histopathological analysis will be required to definitively conclude GLPSGLT topical's effects on skin structure.

A full review of the body of the literature evidence behind each ingredient in GLPSGLT is beyond the scope of this paper. The rationale behind the main active ingredients is described below.

Carotenoid byproducts are a well-known and well-established ingredient in topical formulations, typically prescribed to address signs of aging. In a previous clinical study in the senior author's clinical practice using the GRS as a primary endpoint on 10 (*n* = 10) patients, a topical using a proprietary complex form of Vitamin A as a primary active ingredient led to clinically significant improvements in 13 of 13 domains. Notably, 95% of patients showed improvement to static wrinkles, and 95% demonstrated improvement to surface roughness.^17^ Additionally, although very few of the patients in the referenced study presented with severe elasticity loss at baseline, 100% experienced improvement in their skin elasticity at the conclusion of the study.^17^ These findings demonstrated that Vitamin A as a primary active showed significant promise for addressing GLP-1 RA–induced accelerated signs of aging and loss of elasticity. This premise led to the formulation of a proprietary, first-of-its-kind bioactive soluble Vitamin A derivative in a novel peptide matrix for enhanced dermal availability and decreased negative inflammation.

Retinoids also improve several aspects of skin health and function thought to be negatively influenced by GLP-1 RA use. Vitamin A acts on retinoic acid receptors and retinoid X receptors in keratinocytes, which promotes cell renewal and keratinocyte proliferation through nuclear receptor–mediated gene transcription.^18^ Specifically, Vitamin A increases proliferation of keratinocytes in the epidermis, leading to epidermal thickening, whereas GLP-1 RAs suppress keratinocyte proliferation and lead to epidermal thinning.^4,19,20^ Retinoids influence keratinocyte differentiation, stimulating keratinocytes to develop properly into cells needed for proper skin barrier function. HaCaT (human epidermal keratinocyte) evaluation with GLP-1 use impairs keratinocyte inflammatory signal by activating adenosine monophosphate-activated protein kinase and restraining macrophage migration, thus impairing apoptosis and the natural pathway of keratinocyte repair.^6^ In addition, transforming growth factor beta 1 behavior is modified in ways that appear to overlap what is seen in glucocorticoid therapy.^21^ With continual use, retinoids are known to strengthen the protective function of the epidermis, restrain transepidermal water loss, and protect collagen against degradation.^18^ Because GLP-1 RAs are thought to decrease skin tensile strength and barrier function by thinning the epidermis—and reduce skin elasticity because of decreased barrier function leading to collagen and elastin degradation—bioavailable peptide and Vitamin A derivatives lead to the promotion of a healthy skin barrier and protective effects against collagen and elastin degradation, allowing it to act as a primary active in the GLPSGLT formula.

Additional actives in the GLPSGLT formula include palmitoyl oligopeptide and palmitoyl tetrapeptide. Peptides are short chains of amino acids and are the building blocks of collagen and elastin. Both palmitoyl oligopeptide and palmitoyl tetrapeptide have been demonstrated to improve skin elasticity, as well as the appearance of fine lines and wrinkles, when used in combination topical formulations.^22^ Palmitoyl oligopeptide and palmitoyl tetrapeptide provide demonstrable evidence of elasticity and aesthetic improvements counter to GLP-1 RA's elasticity-reducing and accelerative effects on aging.

Strengths of this study include excellent retention as well as the inclusion of a wide range of patient skin types in a relatively small sample size (*n* = 7) which makes for more informative study results. The use of a split-face design allowed for direct comparison between study topical and placebo-treated skin, allowing for more conclusive analysis of GLPSGLT topical's effects. Also positive was GLPSGLT topical's limited side-effect profile, indicating broad tolerance in a cohort of patients with compromised skin barrier strength. The results of this study indicate that the novel GLPSGLT topical formulation may help reverse skin changes associated with GLP-1 and SGLT-2 use, including reduced elasticity, surface roughness, and impaired hydration. Patients in this study were on GLP-1 RA therapy for as little as 6 months. In the senior author's clinical practice, patients outside of this study population have noted changes to their skin with the use of GLP-1 RAs in as little as 3 months with 10 pound weight loss. Without timely intervention, these structural and functional skin changes may become long-standing or potentially permanent, reinforcing the need for early treatment and preventative care in patients using these medications.

Limitations include a small cohort of patients (*n* = 7). Although the inclusion of blinded third-party photographic review and a patient-satisfaction survey with questions overlapping with the domains of the GRS assessment increases the rigor of the study beyond what would be achievable with the GRS alone, the authors recognize that these assessments are subjective, and like all subjective measures, may be prone to individual interpretation and bias. Evidence from this study suggests, with statistical significance, that there was an improvement to imbalance and asymmetry in all patients. Although “imbalance” is considered a morphologic domain and refers to the profile (“asymmetry” refers to the frontal view of the face), this term may be somewhat unclear to patients, and the degree of change observed in this domain, as well as the larger confidence interval, may be reflective of some patients’ assessing balanced skin tone rather than balance of facial anatomic structures, or may be reflective of patients’ assessing the magnitude of aesthetic skin changes experienced between the treatment and placebo sides of the face because of the split-face study design. Inclusion of an objective scale or outcome measure would strengthen study findings. The cohort of patients in this study included only 3 Fitzpatrick skin types, so future studies should be inclusive of all skin types.

Although there is published proof of histological change to the epidermis, we acknowledge the limitation of not including histology. Furthermore, this study would have benefited from histopathological skin analysis to assess structural skin changes in GLP-1 analog–treated and SGLT analog–treated patients before and after treatment with GLPSGLT topical. Because of the split-face study design, an ethical concern was raised around biopsy of the placebo-treated skin by the IRB. In future studies, histological analysis will be employed to make conclusive statements about the degree of structural skin changes, including the presence of collagen bundles in the skin before and after treatment with GLPSGLT. This study would have also benefited from expanding the inclusion criteria to corticosteroid users experiencing skin atrophy. Given the potential keratinocyte-suppressing mechanisms described for GLP-1 RAs and the keratinocyte-stimulating effects of GLPSGLT topical, this study would have benefited from additionally examining patients with corticosteroid skin thinning, a more well-established cause of keratinocyte-suppression-related skin atrophy. Last, the safety and tolerability described here are short-term (42 days) and do not necessarily reflect long-term safety and efficacy.

Although the current study focuses on the adverse dermatologic effects of GLP-1 RAs, it is important to note their emerging role in modulating inflammation. GLP-1s have demonstrated anti-inflammatory actions in both in vitro and in vivo models, suggesting potential therapeutic utility in conditions such as psoriasis, keloid scarring, and other chronic inflammatory skin diseases. To fully understand the dual nature of GLP-1s—both their beneficial and adverse effects—future studies should incorporate dermatopathological assessments and long-term clinical follow-ups across a range of inflammatory and structural skin disorders.

GLPSGLT was formulated specifically to address the growing concern for skin changes observed in GLP-1 analog–treated and SGLT analog–treated patients. This study supports a role for this formulation as a supplementary at-home treatment for both GLP analog and SGLT users to address related losses of elasticity and firmness, in addition to a wide range of other age-related aesthetic concerns across multiple skin types.

## CONCLUSIONS

In this study, GLPSGLT topical serum demonstrated statistically significant improvement across all 13 domains of the GRS in GLP-1– and SGLT-2–treated patients when applied twice daily for 42 days. Improvements were observed as early as Day 21 and continued through Day 42, with the treated side outperforming the placebo in all measured categories. Independent photographic review confirmed visible enhancement in key domains, such as surface roughness, pigmentation, and static wrinkles. Patients also reported high satisfaction and no significant adverse effects, supporting the serum's safety and efficacy over the 6-week study period.

## Supplementary Material

ojaf030_Supplementary_Data
