# Supplementary material for: A Single-Center, Blinded, Placebo-Controlled Study Evaluating Cosmetic Efficacy and Safety of a Novel Topical GLPSGLT in Glucagon-Like Peptide-1 Analog-Treated Patients
Source: Aesthet Surg J Open Forum. 2025 Apr 25;7:ojaf030. doi: 10.1093/asjof/ojaf030 (PMC12094012; doi:10.1093/asjof/ojaf030)
Supplement: ojaf030_Supplementary_Data [file ojaf030_supplementary_data.docx]

**APPENDIX** A **– SUBJECT QUESTIONNAIRE FOR DAYS 21 AND 42**

**Date:__ */*__ (mm/dd)**

1. Overall, I am very satisfied with the results from the topical labeled “LEFT” after 3 weeks (21 days)

❏ Strongly Agree (5)

❏ Agree (4)

❏ Neutral (3)

❏ Disagree (2)

❏ Strongly Disagree (1)

2. Overall, I am very satisfied with the results from the topical labeled “RIGHT” after 3 weeks (21 days)

❏ Strongly Agree (5)

❏ Agree (4)

❏ Neutral (3)

❏ Disagree (2)

❏ Strongly Disagree (1)

3. I felt that there was a visible reduction in the fine lines and wrinkles on the **LEFT** side of my face after 3 weeks (21 days)

❏ Strongly Agree (5)

❏ Agree (4)

❏ Neutral (3)

❏ Disagree (2)

❏ Strongly Disagree (1)

4. I felt that there was a visible reduction in the fine lines and wrinkles on the **RIGHT** side of my face after 3 weeks (21 days)

❏ Strongly Agree (5)

❏ Agree (4)

❏ Neutral (3)

❏ Disagree (2)

❏ Strongly Disagree (1)

5. The study topical labeled “LEFT” made me feel more confident in the physical appearance of the left side of my face after 3 weeks (21 days)

❏ Strongly Agree (5)

❏ Agree (4)

❏ Neutral (3)

❏ Disagree (2)

❏ Strongly Disagree (1)

6. The study topical labeled “RIGHT” made me feel more confident in the physical appearance of the right side of my face after 3 weeks (21 days)

❏ Strongly Agree (5)

❏ Agree (4)

❏ Neutral (3)

❏ Disagree (2)

❏ Strongly Disagree (1)

7. I felt there was a significant improvement in the texture/smoothness of the skin treated

with the serum labeled “LEFT” after 3 weeks (21 days)

❏ Strongly Agree (5)

❏ Agree (4)

❏ Neutral (3)

❏ Disagree (2)

❏ Strongly Disagree (1)

8. I felt there was a significant improvement in the texture/smoothness of the skin treated

with the serum labeled “RIGHT” after 3 weeks (21 days)

❏ Strongly Agree (5)

❏ Agree (4)

❏ Neutral (3)

❏ Disagree (2)

❏ Strongly Disagree (1)

9. I felt there was a significant improvement in my pore size on the skin treated with the

serum labeled “LEFT” after 3 weeks (21 days)

❏ Strongly Agree (5)

❏ Agree (4)

❏ Neutral (3)

❏ Disagree (2)

❏ Strongly Disagree (1)

10. I felt there was a significant improvement in my pore size on the skin treated with the

serum labeled “RIGHT” after 3 weeks (21 days)

❏ Strongly Agree (5)

❏ Agree (4)

❏ Neutral (3)

❏ Disagree (2)

❏ Strongly Disagree (1)

11. I felt there was a significant improvement in the firmness of my skin treated with the

serum labeled “LEFT” after 3 weeks (21 days)

❏ Strongly Agree (5)

❏ Agree (4)

❏ Neutral (3)

❏ Disagree (2)

❏ Strongly Disagree (1)

12. I felt there was a significant improvement in the firmness of my skin treated with the

serum labeled “RIGHT” after 3 weeks (21 days)

❏ Strongly Agree (5)

❏ Agree (4)

❏ Neutral (3)

❏ Disagree (2)

❏ Strongly Disagree (1)

13. I felt there was a significant improvement in the radiance/luminosity of my skin after

using the serum labeled “LEFT” for 3 weeks (21 days)

❏ Strongly Agree (5)

❏ Agree (4)

❏ Neutral (3)

❏ Disagree (2)

❏ Strongly Disagree (1)

14. I felt there was a significant improvement in the radiance/luminosity of my skin after

using the serum labeled “RIGHT” for 3 weeks (21 days)

❏ Strongly Agree (5)

❏ Agree (4)

❏ Neutral (3)

❏ Disagree (2)

❏ Strongly Disagree (1)

15. I felt there was a significant improvement in my skin tone and evenness after using

the serum labeled “LEFT” for 3 weeks (21 days)

❏ Strongly Agree (5)

❏ Agree (4)

❏ Neutral (3)

❏ Disagree (2)

❏ Strongly Disagree (1)

16. I felt there was a significant improvement in my skin tone and evenness after using

the serum labeled “RIGHT” for 3 weeks (21 days)

❏ Strongly Agree (5)

❏ Agree (4)

❏ Neutral (3)

❏ Disagree (2)

❏ Strongly Disagree (1)

17. I felt there was a significant improvement in my skin moisture after using the serum labeled “LEFT” for 3 weeks (21 days)

❏ Strongly Agree (5)

❏ Agree (4)

❏ Neutral (3)

❏ Disagree (2)

❏ Strongly Disagree (1)

18. I felt there was a significant improvement in my skin moisture after using the serum labeled “RIGHT” for 3 weeks (21 days)

❏ Strongly Agree (5)

❏ Agree (4)

❏ Neutral (3)

❏ Disagree (2)

❏ Strongly Disagree (1)

19. The serum labeled “LEFT” felt comfortable and soothing on my skin

❏ Strongly Agree (5)

❏ Agree (4)

❏ Neutral (3)

❏ Disagree (2)

❏ Strongly Disagree (1)

20. The serum labeled “RIGHT” felt comfortable and soothing on my skin

❏ Strongly Agree (5)

❏ Agree (4)

❏ Neutral (3)

❏ Disagree (2)

❏ Strongly Disagree (1)

21. The serum labeled “LEFT” is gentle enough for everyday use

❏ Strongly Agree (5)

❏ Agree (4)

❏ Neutral (3)

❏ Disagree (2)

❏ Strongly Disagree (1)

22. The serum labeled “RIGHT” is gentle enough for everyday use

❏ Strongly Agree (5)

❏ Agree (4)

❏ Neutral (3)

❏ Disagree (2)

❏ Strongly Disagree (1)

23. I would recommend the serum labeled “LEFT” to family and friends

❏ Strongly Agree (5)

❏ Agree (4)

❏ Neutral (3)

❏ Disagree (2)

❏ Strongly Disagree (1)

24.I would recommend the serum labeled “RIGHT” to family and friends

❏ Strongly Agree (5)

❏ Agree (4)

❏ Neutral (3)

❏ Disagree (2)

❏ Strongly Disagree (1)

25. Have you noticed any other aesthetic effects of the serum labeled “LEFT” over the past 3 weeks (21 days) of applying the product?

Other Comments

26. Have you noticed any other aesthetic effects of the serum labeled “RIGHT” over the past 3 weeks (21 days) of applying the product?

Other Comments:
